# Influence of social networks and environmental factors on older adults’ regular walking

**DOI:** 10.3389/fpubh.2025.1554148

**Published:** 2025-04-11

**Authors:** Hwajun Kim, Young Ko

**Affiliations:** ^1^Health Policy and Management, College of Medicine, Seoul National University, Seoul, Republic of Korea; ^2^College of Nursing, Gachon University, Incheon, Republic of Korea; ^3^Gachon Biomedical Research Institute, Gachon University Gil Medical Center, Incheon, Republic of Korea

**Keywords:** older adults, walking, environment, social factors, neighborhood

## Abstract

**Introduction:**

Few studies have confirmed the influence of social networks and environmental factors on the regular walking of older adults in the community. This study aimed to identify factors influencing regular walking, focusing on social networks and the walkability of the neighborhood environment.

**Methods:**

This study is a secondary analysis of a cross-sectional survey conducted with 840 community-dwelling older adults. Multiple logistic regressions were performed to determine the factors influencing regular walking.

**Results:**

Older women are 1.58 times more likely to walk regularly than men. For older men, the likelihood of regular walking increased 1.56 times as their frequency of contact with friends and neighbors increased. For older women, the probability of regular walking increased by 1.39 times when street connectivity improved. Habitual walking probabilities were lowered by 1.45 times for older women when the terrain was hilly.

**Discussion:**

Health care providers should consider their social networks and environmental factors while developing strategies to promote regular walking in older adults.

## 1 Introduction

Regular walking, an aerobic exercise that consumes calories and provides cardiopulmonary benefits with reduced strain on the joints, is easily adapted by even older adults (1). Regular physical activity helps older adults maintain healthy and independent lifestyles; it also aids in preventing cancer, reduces risk factors for chronic diseases, promotes musculoskeletal health, decreases the threat of mental health problems—including depression, stress, and anxiety (2)—and reduces mortality (3). However, only 39.9% of the older adults in Korea walked regularly in 2019 (4); older adults avoid walking because they fear falling, lack time, lack energy, and display low willpower (5).

Previous studies on factors influencing walking by older adults have reported the association of personal factors such as demographic characteristics, socioeconomic conditions, and physical, mental, and cognitive attributes with walking (6–9). Researchers found that gender and age significantly influence walking, with women reporting higher participation rates for recreational walking than men, and older adults reporting higher rates than younger age groups (6). Adults with lower educational qualifications and low incomes were more likely to report the absence of recreational walking than their counterparts who were better educated and were socioeconomically higher in status (7, 8). Researchers found that health-related factors such as high levels of depression, perceptions of poor health, and fear of falling adversely affect walking by older adults (8, 9).

**Figure 1:**
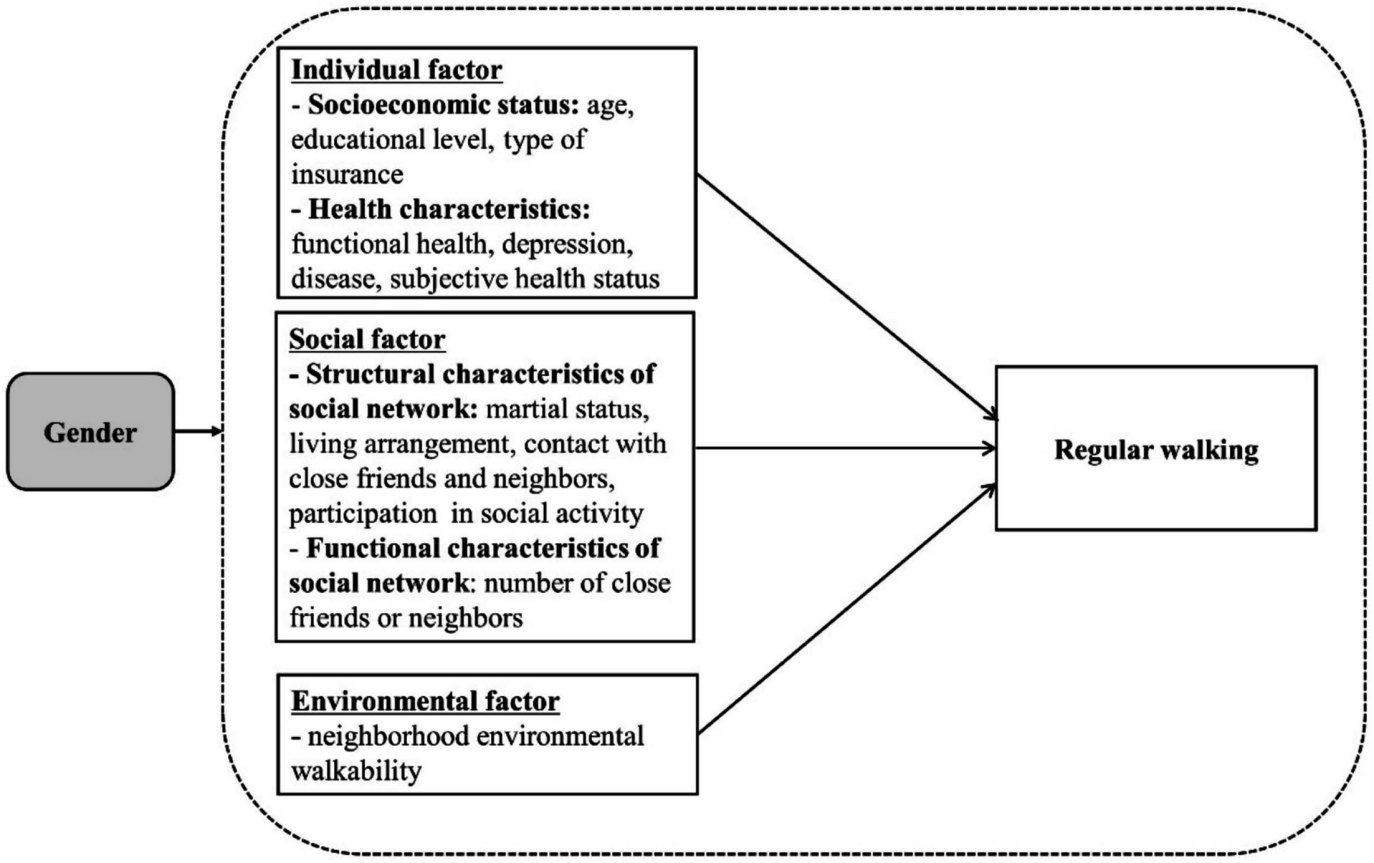
Study framework.

Studies focusing on individual factors influencing the physical activity of older adults revealed the small and short-lived effects of interventions (10, 11). Thus, in addition to individual factors, subsequent investigations have attempted to examine the supplemental impact of environmental and social factors on physical activity (12). Social factors such as social support and social activity are important for habitual exercise (9). Social networks discharge the crucial function of maintaining health and represent a social factor. Social networks are defined as quantifiable relationships between individuals, families, groups, or corporations bonded by common interests, goals, or needs (13). According to a previous study, vulnerable groups exhibited discrete health conditions depending on the extent of their social networks (14). Previous studies have yielded inconsistent results about the impact of social factors like support (9, 15, 16), bonding (8), and interactions (17, 18) on older adults’ walking. This finding implies that the span of social networks may influence walking among older adults. Environmental factors also pivotally influence walking by older adults (19–21). However, the results of two reviews published in 2010 and 2016 were inconsistent with respect to the moderating effects of gender and age on the relationship between the neighborhood environment and walking (22, 23). In addition, environmental factors influencing walking differ by country (24) and gender (25). Biological characteristics affecting health, socioeconomic factors, material resources, and gender roles explain the differences in health behaviors and health status between men and women. Gender is one of main demographic factor explaining these differences (26). Many studies only consider gender as one of the factors influencing health status (27). Therefore, the study aimed to identify gender-specific features that influence regular walking, focusing specifically on social networks and the walkability of the neighborhood environment (Figure 1). It identified gender differences in the rates of regular walking, social networks, and the walkability of the neighborhood environment. The study also identified the factors influencing regular walking, focusing on social networks and the walkability of the neighborhood environment.

We hypothesized that not only individual factors, but also social and environmental factors would be associated with regular walking. We hypothesized that factors related to walking might be different for older women and older men. We assumed that not only individual factors but also social and environmental factors would be related to walking. We also assumed that factors related to walking might be different for older women and older men.

## 2 Materials and methods

### 2.1 Study design

This study represented a secondary analysis of data obtained from a previously conducted cross-sectional exploration of the factors influencing the homebound condition of older adults living in a community (28). The original study was conducted after receiving approval from the institutional review board of the institution to which the researchers were affiliated.

### 2.2 Data and participants

**Figure 2:**
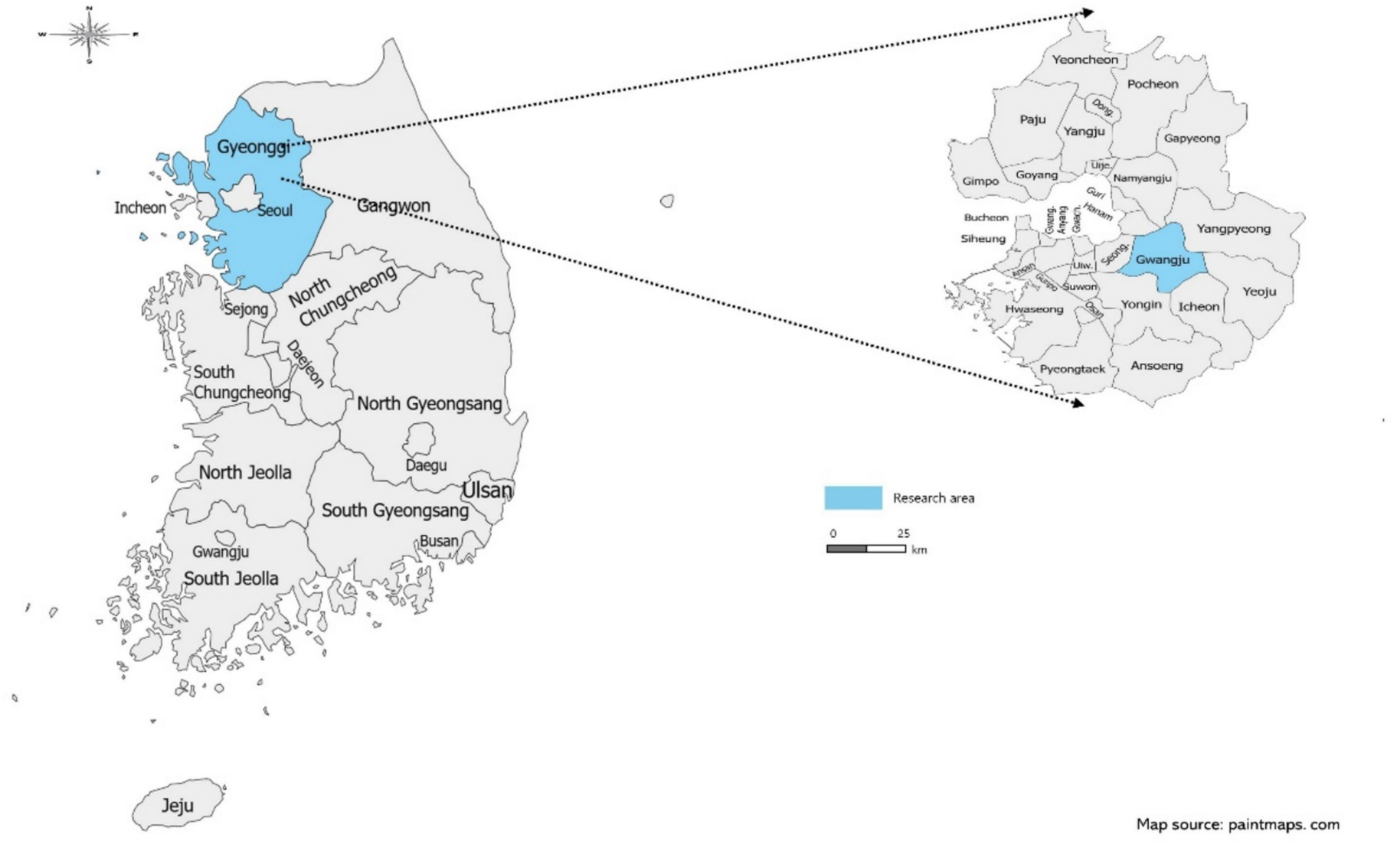
Research area.

The participants of this study were older adults living in the community. We informed the participants of the purpose and contents of the study. The respondents who registered for the study at the public health center in Gwangju City in Gyeonggi-do in South Korea and others living in those areas (Figure 2) were informed about the purpose and contents of the study. Those who desired to participate in the study were recruited through convenience sampling. The current study analyzed data from 840 respondents aged 60 years or older. We predetermined the sample size because this study is a secondary analysis. Multivariate logistic regression analysis was performed to address Aim 2. Based on the recommendation of Peduzzi et al. (29), we calculated the sample size to address Aim 2 via logistic regression analysis: Let *p* represent the smallest proportion of cases in the population, and let k represent the number of independent variables in the logistic regression model. The minimum sample size is *N* = 10 k/p (29). Based on this, the current study necessitated 690 participants, with *p* = 36.2% representing the proportion of men who do not walk regularly, and *k* = 25. Therefore, the predetermined sample size was adequate for the achievement of Aim 2.

### 2.3 Measurement

#### 2.3.1 Social networks

Social networks, as a social factor, were divided into structural and functional characteristics of social networks. The data on marital status, living arrangements, frequency of contact with close friends and neighbors, and frequency of participation in social activities were assessed to identify the structural characteristics of social networks. Marital status was categorized as married/partnered or single/separated/divorced. Living arrangements were classified as living alone or living with others. The frequencies pertaining to contact with close friends and neighbors and participation in a social activity were queried on a 4-point scale (1 = none or almost none, 2 = 1–3 times a month, 3 = more than once a week, 4 = almost every day).

The number of close friends or neighbors and the extent of social support were assessed to ascertain functional characteristics of social networks. This study used Park’s (30) social support scale, which consists of 15 items that measure emotional, cognitive, and material support on a 5-point Likert-like scale. Scores were calculated as the average value of the items, and higher scores indicated greater social support. The scale reliability was computed at 0.979 for this study (30).

#### 2.3.2 Neighborhood environment walkability

The abbreviated version of the neighborhood environment walkability scale (NEWS-A) was employed to evaluate the walking environment (31). Initially, this instrument included 98 items (31); subsequent modifications and revisions resulted in an abbreviated tool (54 items) that demonstrated both construct and criterion validity (32). The validity and reliability of the Korean translation of NEWS-A were also confirmed (25). NEWS-A comprises 54 items arranged into ten sub-domains. The number of items, calculation method, and definitions for the sub-domains are shown in Table 1.

**Table 1:** Sub-domains of neighborhood environment walkability.

Sub-domains	Number of items: calculation	Scale	Score range	Definition of score (association with walkability)
Residential density	6: item 1 + (12*item 2) + (10* item 23) + (25* item 24) + (50* item 25) + (75* item 26)	5-point scale (1 = not at all to 5 very much agree)	173–865	+
Land-use mix-diversity	18: averaging, inverse conversion of all items	5-point scale (1 = 1–5 min, 2 = 6–10 min, 3 = 11–20 min, 4 = 21–30 min, 5 = 31 min or more and I do not know)	1–5	+
Land-use mix-access	3: averaging	4-point scale (1 = strongly disagree to 4 = strongly agree)	1–4	+
Street connectivity	2: averaging	The same as above	1–4	+
Places for walking and cycling	6: averaging	The same as above	1–4	+
Aesthetics	4: averaging	The same as above	1–4	−
Traffic hazards	3: averaging, inverse conversion of the second question among three	The same as above	1–4	−
Crime	3: averaging	The same as above	1–4	−
Lack of parking	1: averaging	The same as above	1–4	−
Lack of cul-de-sacs	1: averaging	The same as above	1–4	+
Physical barrier	1: averaging	The same as above	1–4	−

+ = higher scores indicate greater walkability, − = lower scores indicate greater walkability.

#### 2.3.3 Regular walking

Regular walking was defined by this study in accordance with the criteria stipulated by the Korea National Health and National Examination Survey: “days of more than 10 min of walking in the last week” and “walking more than 30 min per day” for 5 or more days a week (more than 150 min a week) (33).

#### 2.3.4 Covariates

The general characteristics of respondents included gender, age, educational level, and type of health insurance. Types of health insurance were classified in terms of the ownership of National Health Insurance or Medicaid. Health-related characteristics probed by this study included dependence on instrumental activities of daily living (IADL), depression, subjective health status, and the number of chronic diseases. This study measured IADL using a Korean instrumental activities of daily living tool, which Won and colleagues (34) adapted from Lawton et al.’s original instrument (35). We classified the case as “dependent” if any of the 10 items received a response of “partial or complete assistance.” Upon marking all items as “completely independent,” we classified the case as “independent.”

We used Kee’s short-form geriatric depression scale to measure depression (36). This 2-point scale encompasses 15 questions, and scores can range from a minimum of 0 to a maximum of 15; tallies of 0–4 indicate normal and 5–15 signify depression. Kee’s study calculated Cronbach’s ⍺ at 0.88, and the reliability of this scale for the present study was computed Kuder–Richardson Formula 20 (KR-20) coefficient as 0.960. The existence of chronic diseases was determined via doctor-diagnosed hypertension, diabetes, stroke, cancer, arthritis, urinary incontinence, heart disease, chronic respiratory disease, or other ailments, and the number of diagnosed diseases was calculated for each respondent. Subjective health status was marked on a 5-point scale (1 = very bad, 5 = very good) in answer to the question, “How would you describe your health in general?”.

### 2.4 Data collection

The institutional review board, to which the first researcher is affiliated, approved the original protocol to identify the factors influencing the condition of older adults homebound [1044396-201909-HR-174-01]. Officers from K City’s public health center informed the older adults living in the community about the study. We recruited participants who wished to engage with this study. We collected data via structured questionnaires from participants who could communicate and gave their written consent to participate in the research project.

### 2.5 Statistical analysis

We performed all data analyses using SPSS version 25.0 (IBM Corp., Armonk, NY, United States). *p*-values <0.05 were established to indicate statistical significance. A *t*-test or *χ*^2^ test was applied to ascertain significant differences in general characteristics, health-related characteristics, social networks, the walkability of the neighborhood environment, and walking by gender (Aim 1). We used multiple logistic regressions to identify the factors influencing regular walking by gender (Aim 2). The assumptions of logistic regression, including the absence of multicollinearity, were assessed and satisfied (37). In addition, we checked that there was no extreme values or outliers in the continuous predictors (37).

## 3 Results

Table 2 displays the general and health-related characteristics of the participants. Of the total 840 participants, 30.6% were men and 69.4% were women. The average age was 74.10 (±7.90) years and the average duration for education was 5.99 (±4.58) years. Around 80% of the participants owned national health insurance. Approximately 40% were married and 45% lived alone. The prevalence of dependency was 19.2, and 37.1% of the older adults evinced mild or higher levels of depression. The average number of doctor-diagnosed chronic diseases was 1.78 (±1.06).

**Table 2:** Characteristics of the participants (*N* = 840).

Characteristics	Category	Total *n* (%) or mean ± SD	Men *n* (%) or mean ± SD	Women *n* (%) or mean ± SD
Gender	Men	257 (30.6)		
	Women	583 (69.4)		
Age	(years)	73.90 ± 7.50	73.50 ± 6.50	74.10 ± 7.90
Educational level	(years)	7.02 ± 4.85	9.37 ± 4.63	5.99 ± 4.58
Health insurance	National health insurance	677 (80.6)	209 (81.3)	468 (80.3)
	Medi-aid	163 (19.4)	48 (18.7)	115 (19.7)
Disability	Independency in IADL	689 (82.0)	218 (84.8)	471 (80.8)
	Dependency in IADL	151 (18.0)	39 (15.2)	112 (19.2)
Depressive symptoms	Normal	556 (66.2)	189 (73.5)	367 (63.0)
	Mild	206 (24.5)	43 (16.7)	163 (28.0)
	Severe	78 (9.3)	25 (9.7)	53 (9.1)
	(Score)	4.37 ± 3.48	3.83 ± 3.56	4.61 ± 3.42
Number of chronic diseases	(Numbers)	1.76 ± 1.08	1.73 ± 1.10	1.78 ± 1.06
Subjective health status	(Score) (1–5)	2.96 ± 0.86	3.12 ± 0.91	2.89 ± 0.82
Total		840 (100.0)	257 (100.0)	583 (100.0)

IADL, instrumental activities of daily living.

Table 3 illustrates the characteristics of walking, the walkability of neighborhood environment, and social networks. About 52% of the respondents walked regularly, and 55.7% of men and 63.8% of women walked regularly.

**Table 3:** Regular walking, neighborhood environment walkability, and social network.

Characteristics	Category	Total *n* (%) or mean ± SD (*n* = 840)	Men *n* (%) or mean ± SD (*n* = 257)	Women *n* (%) or mean ± SD (*n* = 583)
Regular walking	Yes	468 (55.7)	164 (63.8)	304 (52.1)
	No	372 (44.3)	93 (36.2)	279 (47.9)
Neighborhood environment walkability				
	Residence density (173–865)^†^	385.54 ± 174.44	384.28 ± 174.37	386.09 ± 174.61
	Land-use mix-diversity (1–5)^†^	2.95 ± 0.90	3.02 ± 0.86	2.91 ± 0.91
	Land-use mix-access (1–4)^†^	2.87 ± 0.87	2.86 ± 0.83	2.87 ± 0.88
	Street connectivity (1–4)^†^	2.64 ± 0.89	2.58 ± 0.87	2.67 ± 0.90
	Infrastructure and safety for walking (1–4)^†^	2.67 ± 0.81	2.69 ± 0.76	2.66 ± 0.83
	Aesthetics (1–4)^†^	2.17 ± 0.82	2.21 ± 0.78	2.15 ± 0.84
	Traffic hazards (1–4)^‡^	1.92 ± 0.45	1.90 ± 0.41	1.93 ± 0.46
	Crime (1–4)^‡^	3.62 ± 0.59	3.66 ± 0.56	3.60 ± 0.60
	Lack of parking (single item: 1–4)^‡^	2.96 ± 0.97	3.00 ± 0.92	2.94 ± 0.98
	Lack of cul-de-sacs (single item: 1–4)^†^	2.47 ± 1.03	2.49 ± 1.00	2.46 ± 1.05
	Hilliness (single item: 1–4)^‡^	3.05 ± 0.85	3.09 ± 0.94	3.04 ± 0.95
	Physical barriers (single item: 1–4)^‡^	3.25 ± 0.85	3.25 ± 0.84	3.25 ± 0.86
Social network				
Marital status	Married/partnered	387 (46.1)	158 (61.5)	229 (39.3)
	Unmarried/separated	453 (53.9)	99 (38.5)	354 (60.7)
Living arrangement	Living alone	350 (41.7)	87 (33.9)	263 (45.1)
	Living with others	490 (58.3)	170 (66.1)	320 (54.9)
Frequency of contact with friends and neighborhood	(1–4)	2.77 ± 1.06	2.61 ± 1.06	2.84 ± 1.05
Frequency of participation in social activities	(1–4)	1.91 ± 1.22	1.93 ± 1.24	1.90 ± 1.20
Number of close friends/neighbors		4.80 ± 5.25	5.25 ± 6.43	4.60 ± 4.63
Social support	(Score) (1–5)	3.61 ± 0.91	3.59 ± 0.99	3.62 ± 0.87

^†Higher scores indicate greater walkability; ‡Lower scores indicate greater walkability.^

Residence density was approximately 385.54 points. Land-use mix diversity, land-use mix access, and infrastructure and safety for walking were neighborhood environment characteristics that increased good walkability, while traffic hazards, lack of parking, and hilliness were neighborhood environment characteristics that decreased walkability. These characteristics were similar for men and women.

In terms of social network characteristics, about 40% were married and 45% lived alone. The percentage of married men was 61.5%, and the percentage of living alone was 33.9%, which was lower than for women. The frequency of contacting friends or neighbors was higher for women than for men, but men participated in social activities more than women. The average number of close people was 5.25 ± 6.43 for men and 4.60 ± 4.63 for women, and social support was similar for men and women at 3.61 out of 5. Approximately 52% of the respondents, comprising 55.7% of men and 63.8% of women, regularly walked. Older women were 1.58 times (1.12–2.23) more likely to walk regularly than men (Table 4).

**Table 4:** Gender differences in regular walking.

Comparison (reference)	Crude ORs (95% CI)	*p*	Adjusted ORs^†^ (95% CI)	*p*
Women (men)	1.62 (1.20–2.19)	0.002	1.58 (1.12–2.23)	0.009

^†^Adjusted as all covariates (age, educational attainment, health insurance, disability, depression, number of chronic diseases, subjective health status, neighborhood environment walkability, and social networks).

**Figure 3:**
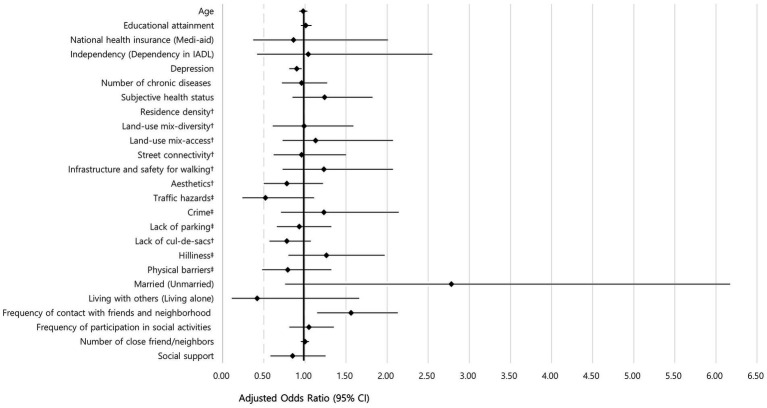
Forest plot regular walking related factors among older men. ^†^Higher scores indicate greater walkability; ^‡^Lower scores indicate greater walkability.

**Figure 4:**
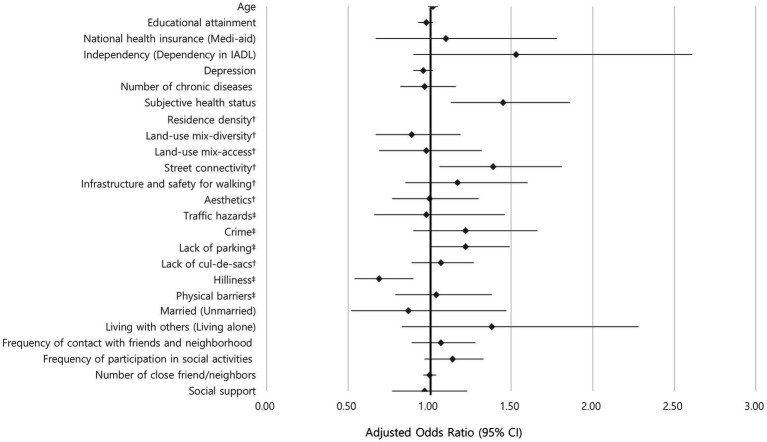
Forest plot regular walking related factors among older women. ^†^Higher scores indicate greater walkability; ^‡^Lower scores indicate greater walkability.

Table 5 shows the results of the multivariate logistic regression analyses performed for factors influencing regular walking among older men and women. The likelihood of regular walking became 1.11 (=1/0.90) (0.81–0.96) times lower for older men as their depression scores increased by one point; their likelihood of regular walking increased 1.56 (1.15–2.13) times as their frequency of contact with friends and neighbors (Table 5 and Figure 3). For older women, the probability of regular walking increased 1.45 (1.13–1.86) times as their subjectively perceived health increased by 1 point; their likelihood of walking regularly also increased by 1.39 (1.06–1.81) times when street connectivity improved. Habitual walking probabilities were lowered by 1.45 (=1/0.69) (0.54–0.90) times for older women when the terrain was hilly (Table 5 and Figure 4).

**Table 5:** Influence of neighborhood environment walkability and social network on regular walking.

Comparison (reference)	Men		Women	
	Adjusted ORs (95% CI)	*p*	Adjusted ORs (95% CI)	*p*
Age	0.98 (0.93–1.03)	0.409	1.02 (0.99–1.05)	0.295
Educational attainment	1.01 (0.95–1.08)	0.680	0.98 (0.93–1.02)	0.301
National health insurance (medi-aid)	0.86 (0.37–2.01)	0.723	1.10 (0.67–1.78)	0.711
Independency (dependency in IADL)	1.04 (0.42–2.55)	0.935	1.53 (0.90–2.61)	0.119
Depression	0.90 (0.81–0.96)	0.039	0.96 (0.90–1.02)	0.194
Number of chronic diseases	0.96 (0.72–1.27)	0.778	0.97 (0.82–1.16)	0.759
Subjective health status	1.24 (0.85–1.82)	0.268	1.45 (1.13–1.86)	0.004
Residence density^†^	1.00 (1.00–1.00)	0.792	1.00 (1.00–1.00)	0.185
Land-use mix-diversity^†^	0.99 (0.61–1.59)	0.955	0.89 (0.67–1.19)	0.438
Land-use mix-access^†^	1.13 (0.73–2.07)	0.659	0.98 (0.69–1.32)	0.763
Street connectivity^†^	0.96 (0.62–1.50)	0.871	1.39 (1.06–1.81)	0.017
Infrastructure and safety for walking^†^	1.23 (0.73–2.07)	0.432	1.17 (0.85–1.60)	0.343
Aesthetics^†^	0.78 (0.50–1.22)	0.272	1.00 (0.77–1.30)	0.984
Traffic hazards^‡^	0.52 (0.24–1.11)	0.092	0.98 (0.66–1.46)	0.935
Crime^‡^	1.23 (0.71–2.14)	0.466	1.22 (0.90–1.66)	0.202
Lack of parking^‡^	0.93 (0.66–1.32)	0.698	1.22 (1.00–1.49)	0.052
Lack of cul-de-sacs^†^	0.78 (0.57–1.07)	0.125	1.07 (0.89–1.27)	0.506
Hilliness^‡^	1.26 (0.80–1.97)	0.323	0.69 (0.54–0.90)	0.005
Physical barriers^‡^	0.79 (0.48–1.32)	0.375	1.04 (0.79–1.38)	0.778
Married (unmarried)	2.78 (0.76–10.17)	0.123	0.87 (0.52–1.47)	0.601
Living with others (living alone)	0.42 (0.11–1.66)	0.215	1.38 (0.83–2.28)	0.213
Frequency of contact with friends and neighborhood	1.56 (1.15–2.13)	0.005	1.07 (0.89–1.28)	0.495
Frequency of participation in social activities	1.05 (0.81–1.35)	0.715	1.14 (0.97–1.33)	0.109
Number of close friend/neighbors	1.00 (0.95–1.05)	0.978	1.00 (0.96–1.04)	0.832
Social support	0.85 (0.58–1.25)	0.401	0.97 (0.77–1.23)	0.799
Nagelkerke *R*^2^	0.183		0.135	
Hosmer and Lemeshow test *χ*^2^(*p*)	6.551(0.586)		3.920(0.864)	

IADL, instrumental activities of daily living.

^†Higher scores indicate greater walkability; ‡Lower scores indicate greater walkability.^

## 4 Discussion

This study attempted to identify factors influencing regular walking for older adults, focusing specifically on the walkability of the neighborhood environment and the social networks. These aspects are discussed below, and their implications are indicated.

First, 55.7% of the participating men and 63.8% of the women walked regularly, and older women were 1.58 times more likely to walk regularly than men. This result is similar to the results reported by previous studies that little difference existed in the total prevalence of walking between older men or women (38, 39), or that the incidence of walking was higher in women than in men (38, 39). The present study’s results of differences between men and women vis-à-vis factors influencing regular walking are consistent with those of previous studies (39). These findings suggest the necessity of examining gender-specific attributes related to regular walking to develop appropriate interventions for men and women.

Second, this study found differences in environmental factors influenced regular walking among men and women. This study found that regular walking was higher in older women when parking lots were adequate, the terrain was less hilly, and there was good street connectivity; however, no related physical environmental characteristics were observed for the older men. The study results are congruent with the results of previous studies that the neighborhood environment exerts a differential effect on men and women vis-à-vis walking (25, 40), and that the neighborhood environment greatly influences walking in women (40).

The study results on the influence of street connectivity and hilliness on regular walking by older Korean women are aligned with the findings reported by studies of adult Korean women (25) and older women living in the USA (24). The improvement of street connectivity by incorporating routes older adults can access and that would allow the older adult to perform their daily activities may increase the walking behavior of women. The results reported by a Finnish two-year follow-up study that hilliness was perceived as an obstacle to walking and that slopes caused walking difficulties in older adults (41) are similar to those revealed by the present study. The fear of falls is higher among older women than in older men (42), notwithstanding the actual incidence of falling. In addition, older men are less likely than women to restrict their activities to prevent falls (43). The findings of this study could be attributed to the fear of falling of older adult women and their perceptions that hilly terrains hinder walking (44).

Traffic and crime safety were found to influence regular walking in previous studies conducted in Nigeria (45) and Brazil (46). Somewhat surprisingly, these factors were irrelevant for the present study. This discrepancy could be caused by regional characteristics. Traffic safety denoted a crucial factor affecting walking by older urban residents dissatisfied with road safety (47). The abovementioned systematic review and meta-analysis did not find traffic/pedestrian safety to represent a major factor affecting exercise, but crime/personal safety was discovered to be a significant element (22). Discrete countries and cities exhibit very different neighborhood environments and lifestyles, and gender differences are noted even in people living in identical communities. Therefore, scrutiny of the contextual meaning of physical environments is necessary for the comprehension of regular walking motivators and deterrents for older adults.

This study reports that older men with higher frequencies of contact with friends and neighbors evince a higher incidence of regular walking. This finding is similar to the result reported by a previous investigation that structural characteristics of social networks are related to physical activity in older adults (48). Another study found a greater influence of network size on mortality through health status in older men individuals than their women counterparts (49); this finding also supports the results of the present study. Previous studies have reported that a “lack of someone to go walking with” denoted a factor triggering a lower likelihood of older adults meeting their physical activity recommendations from the Center for Disease Control and Prevention (9). The maintenance of objective social networks could denote a principal intervention aspect to ameliorate the walking habits of older men.

This study’s finding that the functional characteristics of social networks were not significant for regular walking by both men and women was inconsistent with the results of previous studies. Walking-related human social support was associated with regular walking in older adults (9). Perhaps, this study’s use of tools to examine general social support could explain the variance in results. The discrepancy supports the findings of a systematic literature review confirming that physical activity-related social support was associated with walking, but general social support was not (22).

Depressive symptoms represented the predominant influencing factor for older men and subjective health status was the preeminent element for older women. Previous studies found that subjective health status influenced walking in women (50). Women who perceived their health as poor were more likely to consider health a barrier to physical activity and to exhibit lower self-efficacy than men (51). Mental health issues such as depression and stress have also been reported to influence walking in the older adult (9, 52). The results of this study that socioeconomic factors did not significantly affect regular walking by older adults differ from the findings reported by previous studies (7, 8), which have observed that local socioeconomic characteristics affect walking more than family income levels (22, 53). Individual socioeconomic factors may exercise a minor effect on the walking habits of older adults.

The physical environment studied in the present investigation was similar for the older adult respondents of both biological sexes but the environmental factors influencing regular walking were different for men and women. In addition, objective social relationships were important for the regular walking habit of older men. Therefore, gender differences must be contemplated when interventions are developed to improve regular walking in older adult populations. This study must acknowledge several limitations. First, no causal relationships can be inferred from the data because this study is cross-sectional. Second, this study utilized data obtained solely from residents of an urban–rural complex. Such data could be affected by population density, crime rates, traffic, and other variables. Therefore, varied regions should be taken into account. Third, this study’s environmental scales for living and walking encompassed subjective evaluation factors. Prospective studies should apply objective assessment criteria for physical activity (accelerometer) and environmental elements (geographical information system).

## 5 Conclusion

This study identified the factors influencing regular walking among older adults with a focus on neighborhood walkability and social networks. The findings revealed that older women were more likely to walk regularly than older men, highlighting the importance of considering gender-specific attributes in developing targeted interventions. The finding revealed that structural social network characteristics affected the walking habits of older men and environmental factors such as street connectivity and hilly terrain influenced the walking accomplished by older women. The following recommendations are offered based on the outcomes of this investigation. First, personal, environmental, and social factors exert a differential influence by gender on regular walking. Therefore, gender differences must be considered when strategies are devised to promote regular walking by older adults. Environmental factors played a significant role in promoting walking behavior, particularly among older women. Elements such as less hilly terrain and better street connectivity were associated with higher walking rates in women. These results emphasize the need for urban planning that enhances pedestrian-friendly environments, particularly for older women. Second, older men will enhance their regular walking habits through the maintenance and improvement of objective social networks. However, the functional aspects of social networks did not significantly affect walking for either gender, differing from previous studies. Additionally, mental and physical health factors influenced walking patterns differently, with depressive symptoms affecting men and subjective health status playing a critical role for women. Third, the results of this study should be revalidated using objective tools to measure social networks, physical environments, and walking. In conclusion, this study highlights the importance of gender-sensitive approaches in designing interventions to promote regular walking among older adults. A better understanding of environmental and social influences can contribute to the development of policies and urban planning strategies that encourage walking and improve overall well-being in aging populations.

## Data Availability

The raw data supporting the conclusions of this article will be made available by the authors, without undue reservation.
